# Nature- based interventions. The key role of variables: nature connectedness and social connectedness

**DOI:** 10.1192/j.eurpsy.2025.1982

**Published:** 2025-08-26

**Authors:** M. Gawrych, M. W. Romaniuk

**Affiliations:** 1 Institute of Psychology; 2 The Maria Grzegorzewska University, Warsaw, Poland

## Abstract

**Introduction:**

The nature-based therapeutic perspective includes: green therapy, blue therapy, animal-assisted therapy, and natural landscape therapy. Until now researchers haven’t clearly recognized underlying mechanisms of nature-related mental health well-being.

**Objectives:**

The study aimed to clarify the relationships between experienced nature connectedness (NC) and social connectedness (SC) before and after blue nature based intervention and mental health (WHO-5, PHQ-4)

**Methods:**

Study group included 54 adults (Mean = 30.6; SD 13,2). Participants completed semi-structured questionnaires before and after blue interventions (one week high sea sailing under supervision of two psychologists). The questionaire measured following variables: nature connectedness, social connectedness, well-being, and mental health.

**Results:**

Results showed a significant increase in social and nature connectedness as well as in mental well-being after the blue interventions. Full results will be shown on the e-poster.

**Conclusions:**

The mental health and wellbeing benefits of contact with nature are becoming increasingly recognized in psychology and medicine. The findings support hypothesis that nature connectedness and social connectedness increased after nature based interventions, and therefore they seem to be important factors conected with mental health.

**Disclosure of Interest:**

None Declared

